# Can atopy have a protective effect against cancer?

**DOI:** 10.1371/journal.pone.0226950

**Published:** 2020-02-03

**Authors:** Andrzej Bożek, Jerzy Jarzab, Michal Mielnik, Agnieszka Bogacz, Renata Kozlowska, Dominika Mangold

**Affiliations:** 1 Clinical Department of Internal Disease, Dermatology and Allergology in Zabrze, Medical University of Silesia, Katowice, Poland; 2 Department of Trauma and Orthopaedics, District Trauma and Orthopaedic Hospital, Piekary Sląskie, Poland; King's College London, UNITED KINGDOM

## Abstract

**Background:**

An increased prevalence of allergies and an increased incidence of breast cancer have been observed. The hypothesis that atopy may have a protective effect against the risk of different types of breast cancer was evaluated.

**Methods:**

In this study, 11,101 patients (11,101 women with a mean age of 55.2±14.7 years) with different types of breast cancer were tested for allergies. Allergies were confirmed based on the retrospective analysis of allergy diagnostic procedures in patients who had been previously diagnosed with breast cancer. The retrospective prevalence rates of active allergic diseases, including allergic rhinitis, bronchial asthma and atopic dermatitis, were assessed. All patients were also analyzed for bronchial asthma and allergic rhinitis according to the relevant guidelines. A group of healthy control patients was used for the comparisons.

**Results:**

The women with breast cancer had a significantly lower incidence of IgE-mediated allergic diseases than the controls. The odds ratios (ORs) for allergic rhinitis, atopic dermatitis, and bronchial asthma were 0.61 (95% CI: 0.57–0.73), 0.17 (95% CI: 0.11–0.44), and 0.73 (95% CI: 0.65–0.83), respectively. The mean serum concentrations of total IgE were significantly lower in the study population of women with breast cancer than in the patients of the control group (39.2 ± 26.2 kU/L vs. 108.5 ± 38.5 kU/L; p = 0.002).

**Conclusion:**

Our results suggest that the overall incidence of allergies, especially allergic rhinitis, is lower in patients with certain types of cancer than in individuals who did not have cancer. Further studies are needed to confirm our findings.

## Introduction

Breast cancer is the second most common cancer diagnosed worldwide. Breast cancer is also the most commonly occurring cancer among women (approximately 12% of women worldwide). There are many risk factors for developing breast cancer, for example, the occurrence of mutations in the BRCA1 and BRCA2 genes, hormone therapy during menopause, first menstruation at an early age, late motherhood or no children, older age, and a positive family history of breast cancer [1,2]. The etiology of breast cancer is complex, and although many factors that affect the risk of developing the disease have already been identified, the causes of the disease cannot be determined in the majority of patients [3,4]. Among many potential factors, the possibility of allergies promoting or protecting against cancer still needs verification [5,6,7,8].

The possible relation between atopy and the induction of neoplasms such as breast cancer is still being investigated; however, few studies have focused on this topic. Some studies have reported allergies in patients with different types of neoplasms; however, the determination of a possible causal relationship was ambiguous [9,10,11]. Kozlowska et al. confirmed the association between IgE-mediated allergies and certain less prevalent types of cancers [12]. However, in this study, the subgroup of women with breast cancer was too small, and the whole patient group was too heterogeneous to reach a conclusion about the relationship between the types of cancer and atopy [12].

In a meta-analysis of available data by Wang et al. that analyzed the relationship between atopy and cancer, the authors also present some studies on breast cancer [13]. There were two retrospective studies that demonstrated a nonsignificant increased risk (a relative risk/odds ratio) of breast cancer among women who reported any history of allergy and one prospective study that found that a history of atopy was a risk factor for premenopausal breast cancer [14,15,16]. In contrast, a retrospective study in the USA found no association with breast cancer among women who had a history of allergy [17]. Other authors found that the prevalence of atopy was reduced in breast cancer patients, but a small group of women was analyzed [18]. Wang emphasized that the limitations of these studies were small groups, a lack of consideration of other factors and often no data about how atopy was confirmed [13].

The aim of this study was to test the hypothesis that there is a relationship between atopy and breast cancer in women.

A retrospective method was used to assess the relationships between certain medical events, the occurrence of allergies and breast cancer in this case. This is the first step before for a planned prospective study that could indicate a possible link between breast cancer and atopy.

## Materials and methods

The methods were similar to those of the study by Kozlowska et al. [12]. This retrospective cross-sectional study assessed the history of ever having allergies in women with breast cancer.

The medical data from patients’ charts were obtained from 3 oncological centers, 15 GP centers and a regional allergology clinic, which cooperated together in southern Poland. The study was based on a cooperative program, which was carried out as part of a multicenter study on the monitoring of atopic diseases and acceded to the consent requirements of the bioethics committee at the Medical University of Silesia (KNW/0022/KB1/18/14).

### Patients

The epidemiological structure and socioeconomic distribution of the respondents were matched to the Poland 2017 statistical data. The study population was selected using stratified sampling and included women over 30 years of age. The study population sampling method was designed to provide a population that was representative of the typical age and sex of those with breast cancer in Poland in 2017.

**Fig 1:**
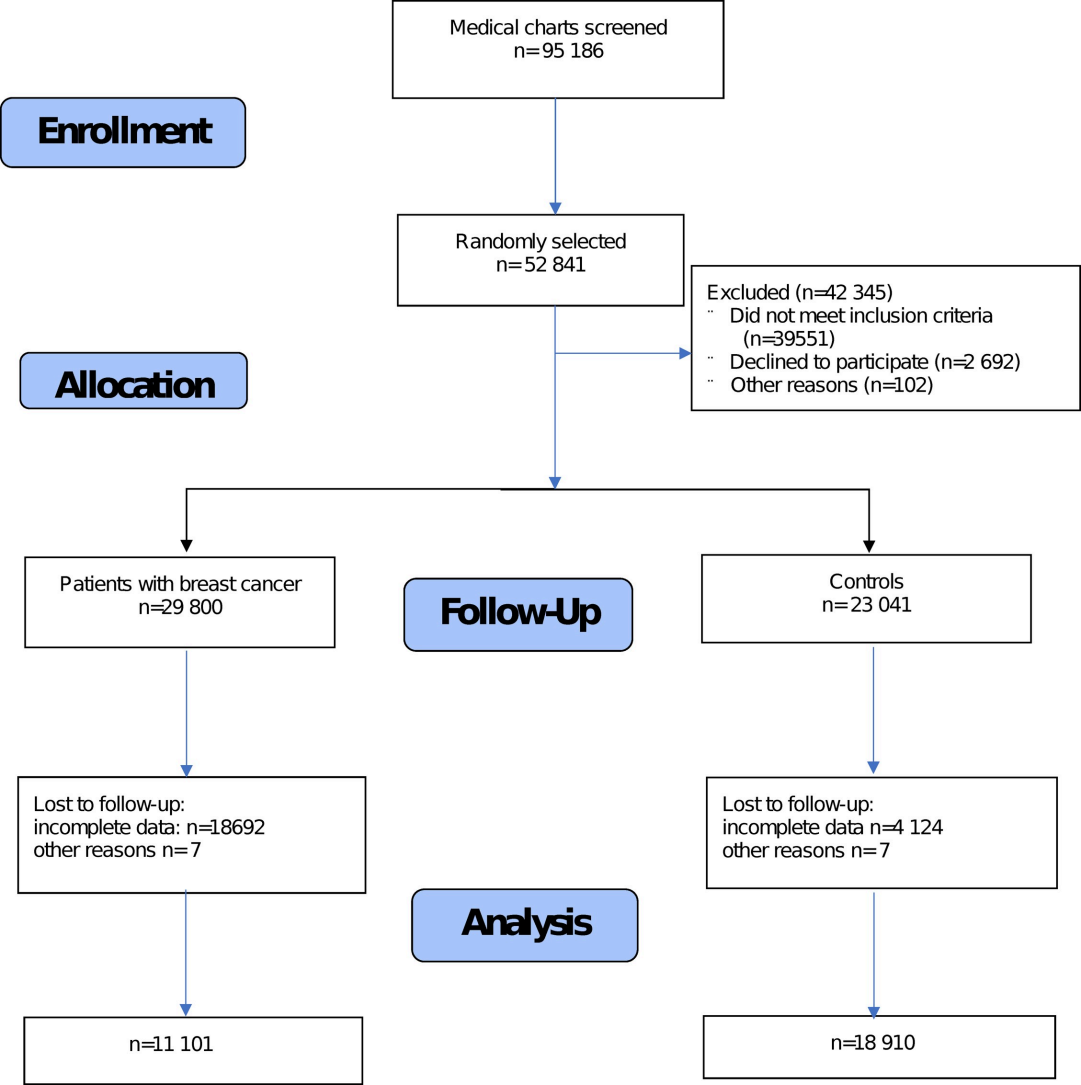
Number of participants who were assessed for eligibility and completed the study.

The patient databases from each site were analyzed simultaneously. In total, 52,308 of a possible 95,186 medical charts were randomly selected and checked. The number of participants that were assessed for eligibility and completed the study is presented in the diagram in Fig 1.

The inclusion criteria for the study group were as follows:

1. Patients were over 30 years of age;
2. Patients had a confirmed breast cancer diagnosis; and
3. Patients underwent basic oncological treatment (i.e., chemotherapy, radiotherapy and/or surgery).

Based on these criteria, 28,800 patients were enrolled.

The exclusion criteria were a lack of detailed documentation and a lack of patient consent. Finally, a group of 11,101 patients comprised of women aged 30 to 84 years was analyzed. The mean time of breast cancer disease duration was 6.5±4.1 years, and the mean time after basic treatment was 2.1±1.4 years at the time of data collection (March-July 2018).

The control group included 18,910 patients (women) who were selected from GP centers and allergological clinics (Fig 1). The inclusion criteria for the control group were as follows:

1. Women over 30 years of age;
2. Individuals without a diagnosis or clinical suspicion of breast cancer.

The distribution of age and sex was similar to that of the study group.

The control group was formed by screening medical records from the medical databases of GPs and regional allergological clinics in the same region of Poland as the main study group.

The same exclusion criteria that were used in the study group were used for the control group.

### Procedures

#### Allergy data

Allergic rhinitis was confirmed based on medical documentation, including laryngological examinations and a history of clinical symptoms according to the ARIA guidelines [13]. Bronchial asthma was confirmed by the documentation of clinical symptoms according to the GINA guidelines and spirometry data and/or positive results from reversibility tests or positive results from methacholine tests, in accordance with the criteria of the ATS and ERS [14]. Atopic dermatitis was confirmed with dermatological examinations and the criteria defined by Hanifin and Rajka [15]. Only active symptoms of allergy were analyzed, wherein active allergy means allergy requiring treatment at the time of analysis.

The measurement of the concentration of total IgE and allergen-specific IgE (sIgE) was performed using a Pharmacia CAP system (FEIA, Pharmacia AB, Sweden). The following specific IgE allergens were analyzed: *D*. *pteronyssinus*, *D*. *farinae*, *Aspergillus fumigatus*, *Alternaria tennis*, *Cladosporium herbarium*, dog, cat, grass mix, birch, alder, hazel, and mugwort. IgE values above 0.35 IU/ml were considered positive.

Skin-prick tests (Allergopharma, Reinbeck, Germany) to the inhalant allergens mentioned above were performed according to previously published guidelines [15].

All types of cancer were previously diagnosed based on clinical symptoms, imaging tests (e.g., X-ray, CT, MRI, PET, endoscopy, and USG), biopsies, serum measurements, cancer markers and the ICD-10 code. The study involved only patients who had received oncological treatment and were undergoing follow-up.

#### Statistics

The data were analyzed with a statistical software package (STATISTICA version 8.1, Statsoft, Cracow, Poland). The parametric descriptive data were compared using Student’s t test or the Mann-Whitney U test. To explore the association between the presence of breast cancer and reports of allergy, asthma, allergic rhinitis, and atopic dermatitis, a multiple logistic regression model was estimated, and the results are presented as the odds ratios (ORs) and 95% confidence intervals (95% CIs).

Cluster analysis was used to reveal hidden structures by grouping patients with similar characteristics into homogenous groups while maximizing the heterogeneity across groups. Such a tool increases the reliability of the observations obtained in a retrospective study, especially when data are from different sources. Moreover, this method is recommended when we expect a rare medical event [19].

In the present study, the program STATISTICA was used to analyze important variables that are described below, and it self-formed models based on the mutual relations of the examined variables. The k-means method, as the most widely used centroid-based clustering algorithm, was performed. It is a statistical tool for searching for dependencies of examined features that were not established for example in retrospective cross-sectional studies. Based on these analyses, 5 different clusters (models) were obtained.

The following patients’ features were tested as variables: low (<30 kU/L), medium (30–100 kU/L) or high (>100 kU/L) concentration of total serum IgE; all of the comorbidities mentioned in Table 1; smoking; living in a rural or an urban area; the presence of an IgE-mediated allergy (allergic rhinitis, bronchial asthma, atopic dermatitis, or separate diseases); and the presence of house dust mite, pollen or animal allergy (indicated by a positive SPT and/or sIgE to house dust mite allergens). All variables were obtained during the collection of data. After forming clusters, the five obtained models were tested according to the proportions of BC and non-BC individuals.

**Table 1:** Characteristics of the study population and control group.

Features	Breast cancer	Control
Women	11 101	18 910
Mean age (years)	48.2±17.7	44.8 ± 9.7
*Ductal carcinoma in situ*	2 099 (18.9%)	-
*Lobular carcinoma in situ*	423 (3.8%)	
*Invasive ductal carcinoma*	6,859 (61.7%)	
*Invasive lobular carcinoma*	865 (7.8%)	
*Adenocystic carcinoma*	281 (2.5%)	
*Metaplastic carcinoma*	133 (1.2%)	
*Micropapillary carcinoma*	97 (0.9%)	
*Mucinous carcinoma*	78 (0.7%)	
*Papillary carcinoma*	76 (0.7%)	
*Tubular carcinoma*	45 (0.4%)	
*Mixed carcinoma*	87 (0.8%)	
*Others*	58 (0.5%)	
*Premenopausal BC*	2 150 (19.4%)	-
*Postmenopausal BC*	8 951 (80.6%)	-
Family oncological history	5 278 (47.5%)	1 878 (9.9%)
Urban area (%)	7 650 (68.9%)	13 238 (70%)
Rural area (%)	3 451 (31.2%)	5 672 (30%)
Current or former smoker	3 105 (30%)	2 463 (13.0%)
Active symptoms of allergy when BC was confirmed	2 056 (18.5%)	5 201 (27.5%)
Allergy including symptoms in the past	2 351 (21.2%)	6 098 (32.2%)
Mean age at onset of allergy symptoms (years)	11.2±7.8	9.1±5.6
Mean time of allergy durations (years)	18.6±17.5	20.1±11.8
Atopic dermatitis	431 (3.8%)	767 (4.1%)
Allergic rhinitis	1 990 (17.9%)	3 740 (19.6%)
Bronchial asthma	897 (8.1%)	2 118 (11.2%)
Diabetes	1 080 (9.7%)	1 323 (7%)
Arterial hypertension	2 045 (18.4%)	2 201 (11.6%)
Overweight (BMI>25)	2788 (25.1%)	4 044 (21.4%)
Heart failure	845 (7.6%)	752 (4%)
COPD	1 028 (9.3%)	1 437 (5.6%)

BMI—body mass index, COPD—chronic obstructive pulmonary disease

This was done to see the possible relationship between the clusters (endotypes) of patients studied and the occurrence of breast cancer. ANOVA or Pearson’s chi square test were used to compare differences between variables in the obtained cluster models.

These endotypes were supposed to show the possible relationship between the various clinical features of the occurrence of allergy, the results of tests, patient lifestyle and the occurrence of breast cancer.

The study was approved by the local ethics committees of the Medical University of Silesia in Poland (KNW/0034/2011). All patients signed an informed consent form.

## Results and discussion

The obtained results revealed a significantly lower prevalence of atopic disease in women with breast cancer compared to controls. However, the mean time of allergy duration and the distribution of the type of atopic diseases were similar between the two groups. The population details are presented in Table 1.

The OR of a clinical manifestation of any active allergy (i.e., allergic rhinitis, conjunctivitis, atopic dermatitis, or bronchial asthma) in women from the study group who were diagnosed with different types of breast cancer was 0.53 (95% CI: 0.43–0.72). The OR for each type of allergy was as follows: allergic rhinitis, 0.61 (95% CI: 0.57–0.73); atopic dermatitis, 0.17 (95% CI: 0.11–0.44); and bronchial asthma, 0.73 (95% CI: 0.65–0.83). The presence of pre- or postmenopausal breast carcinoma did not influence the frequency of allergy. There were no significant differences in the allergen profiles of the analyzed allergic patients in the cancer and control groups. Allergy to mites and grass pollen were predominant in both groups as follows: *D*. *pteronyssinus* (32.1% of the study population with breast cancer vs. 30.5% of the control group, p = 0.21), *D*. *farinae* (32.7% vs. 31.6%, p = 0.14) and grass pollen (26.5% vs. 28.9%, p = 0.32).

The mean serum concentration of total IgE was significantly lower in the study population than in the control group (39.2 ± 26.2 kU/L vs. 108.5 ± 38.5 kU/L; p = 0.002). An inverse correlation between the total IgE concentration and the presence of breast cancer was observed (R = -0.78, the point-biserial correlation coefficient, p = 0.01). Total serum IgE was independent of the type of breast cancer, except for metaplastic carcinoma, where a correlation was observed. The details are presented in Table 2.

**Table 2:** The profile of the types of breast carcinoma, the mean value of total IgE and the risk of allergy (HR).

Breast cancer type (%) in the study population	Mean serum concentration of IgE (kU/L)	OR of IgE-mediated allergy
		(95% CI)
*Ductal carcinoma in situ*	48.31	0.54 (0.42;0.59)
*Lobular carcinoma in situ*	37.19	0.81 (0.72;0.85)
*Invasive ductal carcinoma*	12.wrz	0.81 (0.77;0.94)
*Invasive lobular carcinoma*	67.23	0.76 (0.64;0.88)
*Adenocystic carcinoma*	17.91	0.66 (0.61;0.72)
*Metaplastic carcinoma*	80.12	0.82 (0.74;0.89)
*Micropapillary carcinoma*	67.22	0.74 (0.64;0.8)
*Mucinous carcinoma*	25.19	0.61 (0.52;0.83)
*Papillary carcinoma*	19.91	0.59 (0.47;0.77)
*Tubular carcinoma*	20.89	0.66 (0.51;0.85)
*Mixed carcinoma*	16.wrz	0.82 (0.71;0.93)
*Others*	23.98	0.45 (0.32;0.58)

OR—odds ratio

Cluster analysis

The cluster analysis revealed five models as follows.

Model 1: a low concentration of total IgE, urban area, a high incidence of comorbidities, a low risk of active allergy, and a low risk of atopic dermatitis.

Model 2: a moderate concentration of total IgE, nonsmoking, rural area, and a low risk of allergy.

Model 3: a moderate concentration of IgE, smoking, a high risk of bronchial asthma symptoms, and atopy in family.

Model 4: a low concentration of IgE, a low risk of allergic rhinitis and asthma symptoms, and comorbidities.

Model 5: a low concentration of IgE, nonsmoking, urban area, and an allergy to house dust mites.

The detailed characteristics of the clusters are presented in Table 3.

**Table 3:** The characteristics of the obtained clusters.

Variables	Model 1	Model 2	Model 3	Model 4	Model 5
number of patients	4954	8242	4231	6335	6009
< 35 yrs (%)	1269 (25.6)	980 (11.9)	654 (15.5)	1162(18.3)*	562 (9.4)
≥ 35 yrs (%)	3685 (74.4)	7262 (88.1)	3577 (84.5)	5173 (81.7)	5447 (90.6)
smoking (%)	1045 (21.1)	523 (6.3)*	1309 (30.9)	2002 (31.6)	756 (12.6)
rural area of living (%)	3210 (64.8)	4758 (57.7)	2101 (49.7)	3090 (48.8)	4671 (77.7)
atopy in family (%)	2121 (53)	2654 (29.2)*	2912 (41.5)	1733 (48.2)	1009 (16.8)
serum concentration of total IgE (kU/L) (%)	77 ± 34	188 ± 41*	124 ± 81	101 ± 78	194 ± 83*
<30	3121 (63)	2982 (36.2)	963 (22.8)	3549 (56)	3391 (56.4)
30–100	1287 (26)	3651 (44.3)	2793 (66)	1993 (31.5)	1797 (29.9)
>100	546 (11)	1609 (19.5)	475 (11.4)	793 (12.5)	821 (13.7)
allergic rhinitis (%)	565 (11.4)	909 (11)	593 (14)	215 (3.4)*	714 (11.9)
allergic asthma (%)	472 (9.5)	455 (5.5)	534 (12.6)*	343 (5.4)	298 (5)
atopic dermatitis (%)	32 (0.6)*	134 (1.6)	65 (1.5)	112 (1.8)	102 (1.7)
allergy to HDM (%)	278 (5.6)	351 (4.3)	249 (5.9)	388 (6.1)	704 (11.7)*
allergy to pollen (%)	389 (7.8)	570 (6.9)	259 (6.1)	311 (4.9)	289 (4.8)
allergy to animals (%)	27 (0.5)	39 (0.5)	12 (0.3)	18 (0.3)	13 (0.2)
comorbidities (%)	1515 (30.6)*	842 (10.2)	780 (18.4)	2101 (33)	674 (11.2)
BC diagnosis (%)	3418 (69)*	2885 (35)	2327 (55)	4941 (78)*	2704 (45)

*significant difference between individual model and other models, p<0.05 (ANOVA or Pearson’s chi square test); HDM—house dust mite.

These results confirmed the possible relation between breast cancer diagnoses and some evidence of allergies. Models 2 and 5 show the inverse association between IgE concentration and the presence of breast cancer diagnosis. On the other hand, in models 1 and 4 with a high prevalence of women with breast cancer, the mean concentrations of IgE were relatively lower in comparison to others. However, the other analyzed allergic features, for example, type diseases, did not have a strong association with the occurrence of breast cancer. These results are consistent with those of a previous study from the Kozlowska group [12]. The results obtained here also confirmed that the relationship between atopy and cancer is complex, even in specific subtypes of cancer. As mentioned above, some studies have previously analyzed the influence of atopy on the risk of breast cancer. Vojtechow et al. found no evidence that asthma, hay, fever and other allergies were associated with breast cancer (relative risk (RR) = 0.93) [12]. Taghizadeh et al. observed that patients with positive skin prick tests or IgE had a lower hazard ratio of mortality related to breast cancer (0.57 and 0.48, respectively). However, the authors did not find an overall association between objective allergy markers and cancer in the total population [13]. Skaaby et al. did not find support for such a hypothesis, based on a study in which approximately 15,000 patients were analyzed for the association between atopy and the prevalence of cancer [15]. The author noticed an association between atopy and a lower risk of dying from breast cancer, but not between atopy and cancer in general [16]. Kozlowska et al. also noticed that the serum concentration of the total IgE was inversely correlated with breast cancer diagnoses and that the risk of IgE-mediated allergic disease was low among breast cancer patients. However, there was no correlation between positive results for inhalant allergen-specific IgE testing and the incidence of breast cancer [14]. In this study, the results obtained were similar. Unfortunately, other studies have analyzed somewhat different types of populations, i.e., patients without an initial cancer diagnosis, with different types of cancer and with only self-reported allergy information [17, 18].

The possible mechanism that could explain the inverse correlation between atopy and breast cancer is the lack of hyperreactivity of the immune system in women with this cancer, as observed in allergic subjects. The immune system can recognize and remove neoplastic cells; and sometimes, this role could be organ specific [19].

On the other hand, the results showed a lower risk of IgE allergic diseases in the study population with breast cancer than in the general population. This risk was significantly lower than that observed by some other authors. However, the analyzed population was significantly smaller, and the criteria for an atopic diagnosis were imprecise [20,21,22,23].

The protective role of IgE against carcinogenesis was confirmed by Van Hemelrick et al. [11]. This study highlighted the lack of a simple link between not only atopy and the risk of developing a neoplasm but also between atopy and other chronic diseases [18]. This finding further supports the complexity of this problem.

The cluster analysis revealed that patients with breast cancer dominated clusters 1 and 4, where a low concentration of IgE and a low risk of allergic diseases were found. This finding is consistent with other results showing an inverse correlation between active symptoms of allergic disease and the presence of breast cancer.

This study was limited by some procedural limitations. The assessment of the allergies in patients with cancer diagnoses was complicated and affected by a number of factors. Patients and physicians were typically focused on addressing the underlying disease, i.e., cancer.

The influence of oncologic therapies, such as chemotherapy and radiation therapy, could not be excluded from influencing the results of the allergy tests (i.e., false negatives). There were no analyses for IgE-mediated food allergies in this study because of the relative rarity of these conditions in adults.

Finally, the fact that the control group was obtained from GP sites and the study patients from outpatient oncology clinics, despite having similar ages and being the same sex, could have been an important confounder (resulting from a lack of uniform data and greater attention being paid to patients with cancer, which yielded richer medical documentation).

The authors are aware of the study restrictions. Despite the use of restrictive statistical tools recommended in such studies, the obtained observations should be treated as preliminary. Basing data only on the analysis of documentation results is necessary to eliminate a large group of data that could significantly affect the final results. This study is the starting point for a future prospective study with a similar purpose and assumptions.

## Conclusion

The observed results may indicate that there is an inverse association between the incidence of breast cancer and the low frequency of IgE-mediated allergic diseases. A protective role of IgE in breast cancer is possible. Further studies are needed to confirm our findings.
